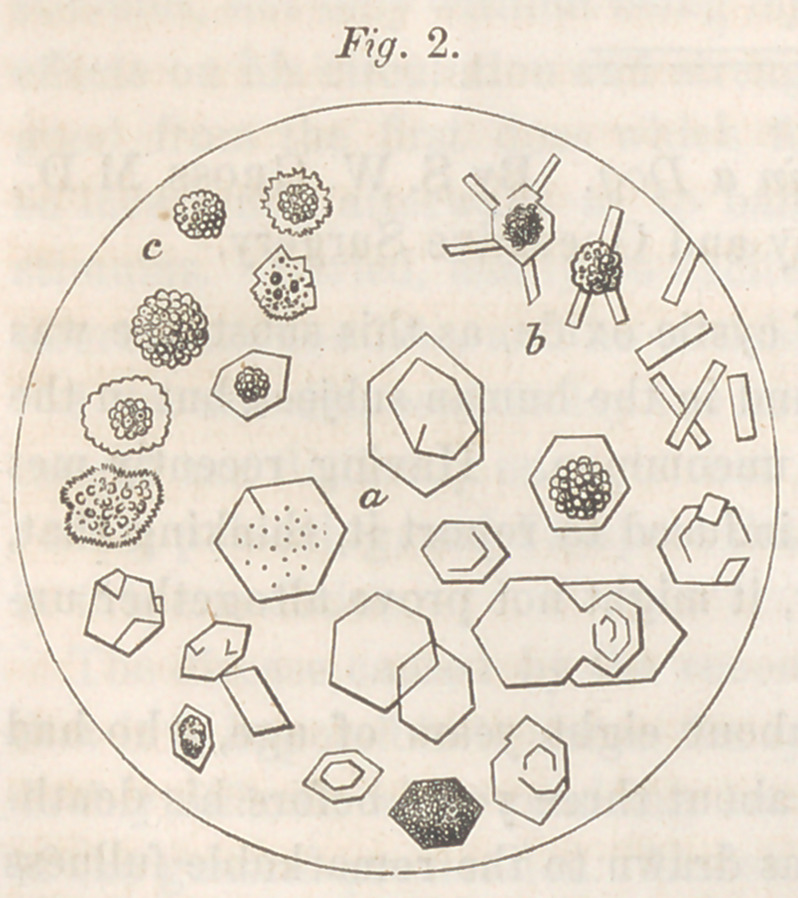# Case of Cystine Calculus in a Dog

**Published:** 1861-03

**Authors:** S. W. Gross

**Affiliations:** Lecturer on Surgical Anatomy and Operative Surgery


### ART. II.—*Case of Cystine Calculus in a Dog.* By S. W. GROSS, M.D., Lecturer on Surgical Anatomy and Operative Surgery.

CALCULI, composed of cystine, or of cystic oxide, as this substance was called by Dr. Wallaston, are rarely found in the human subject, but in the dog their occurrence is by no means uncommon. Having recently met with a case of this kind, I have been induced to report it, thinking that, as an object of comparative pathology, it might not prove altogether uninteresting.

The subject was a Scotch terrier, about eight years of age, who had been struck over his loins with a stick about three years before his death. During August, 1860, my attention was drawn to the remarkable fullness of his perineum, and the very frequent desire which he evinced to make water, the act being somewhat difficult. He soon began to lose his usual sprightliness, and walked with considerable difficulty. Some weeks having elapsed, he showed signs of great suffering, barking and howling in the most piteous manner. The urine generally passed by drops, was frequently bloody; and when he walked, his hind and fore legs were drawn together, his loins were curved and his flanks drawn in. His appetite gradually failed, and he lost flesh. Several weeks before his death, which took place about the middle of December, his gait was that of paralysis, the hind limbs being dragged after him.

*Fig.* 1.

These symptoms pointed unmistakably to the existence of a vesical calculus, and I was therefore not disappointed on opening the body in finding a beautiful stone, the external appearance and dimensions of which are shown in *fig.* 1. It was grasped by the apex of the bladder, which was much contracted, and a portion of its mucous membrane was adherent to the surface of the calculus. The prostate gland was somewhat hypertrophied, but the kidneys and ureters, as well as the other internal organs, were normal.

The calculus was semi-crystalline, and covered with smooth tubercles; its color was that of ordinary beeswax, its weight eighty grains, and specific gravity 1·060. Being easily scraped with the knife, I obtained a sufficient quantity to act upon by chemical tests. It was found to be soluble in sulphuric, nitric, and chlorohydric acids, and in ammonia. Nitric acid being evaporated upon it, a dark-brown residue was left, and under the blow-pipe it was dissipated with a very disagreeable odor. Liebig's test showed very conclusively the presence of sulphur.

*Fig.* 2.

A few drops of liquor ammoniæ being added to the calculous scrapings, and the solution allowed to evaporate slowly on a glass slide, presented under the microscope the characteristic crystals of cystine, shown in *fig.* 2. At *a* are seen hexagonal plates, for the most part transparent; others are opaque in their centres, and some are laminated. At *b* the crystals assume a rectangular form; and at *c* they present themselves as round, or irregular hexagonal masses, crenate at the margins, dark in the centre, and resembling rosettes. The latter form and the six-sided plates existed in the greatest abundance, and very often formed large and confused masses.

## Figures and Tables

**Fig. 1. f1:**
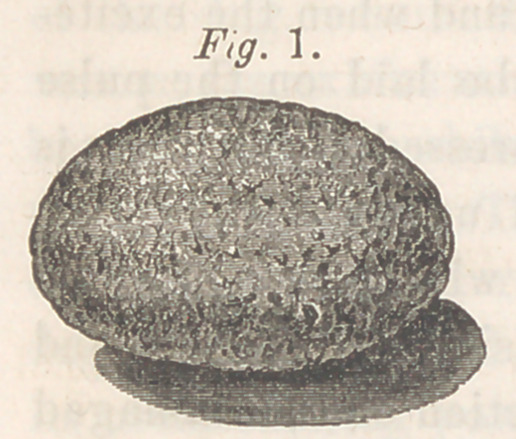


**Fig. 2. f2:**